# Microbiota Composition and Diversity in Weight Loss Population After the Intake of IQP-AE-103 in a Double-Blind, Randomized, Placebo-Controlled Study

**DOI:** 10.3389/fnut.2022.790045

**Published:** 2022-04-28

**Authors:** Li Vern Peng, Jennifer Cooper, Patricia De Costa, Pee Win Chong

**Affiliations:** ^1^InQpharm Group Sdn Bhd, Kuala Lumpur, Malaysia; ^2^Lead Point Solutions, Inc., Provo, UT, United States

**Keywords:** okra, inulin, microbiota, obesity, metabolic syndrome, *Akkermansia muciniphila*

## Abstract

The effect of the novel IQP-AE-103 (proprietary combination of dehydrated okra powder and inulin) on body weight reduction and the association with changes in microbiota composition were investigated in a double-blind, randomized, placebo-controlled trial. A total of seventy-two overweight or moderately obese subjects with a body mass index of ≥25 and <35 kg/m^2^ were randomly allocated to receive IQP-AE-103 or placebo; each group received two IQP-AE-103 or placebo capsules three times daily, respectively. Body weight, body fat, waist circumference, and hip circumference were measured, and fecal samples were collected at baseline and after 12 weeks of intervention. Using 16S rRNA gene sequencing on the fecal samples, the microbiota dissimilarity, diversity, and differences in relative abundance between or within groups were analyzed. At the end of the study, body weight was significantly reduced in the IQP-AE-103 group compared with the placebo group, 5.16 ± 2.39 kg vs. 0.97 ± 2.09 kg (*p* < 0.001). Subjects from the IQP-AE-103 group who achieved a reduction of ≥5% of total body weight from baseline (hereafter referred to as 5% responders or IQP5) had a mean body weight reduction of 6.74 ± 1.94 kg, significantly greater than the placebo group (*p* < 0.001). Using Lefse and statistical analysis, subjects in the IQP-AE-103 group had a significantly lower relative abundance of Firmicutes than the placebo group (*p* < 0.05) after 12 weeks of intervention. The 5% responders from the IQP-AE-103 group had a remarkable 4.6-fold higher relative abundance of *Akkermansia muciniphila* than the placebo group (*p* < 0.05). As the significant differences between groups were only observed post-intervention, the overall differences in microbiota profile suggest that the weight loss in overweight and moderately obese subjects who consumed IQP-AE-103 for 12 weeks is accompanied by a positive change in microbiota composition. These changes might be linked to the beneficial effects of microbiome modulations in alleviating obesity and metabolic syndrome. To the best of our knowledge, we are the first to report over-the-counter (OTC) supplementation that results in both significant changes in weight and favorable shifts on the subject microbiota profile. The trial is registered under ClinicalTrials.gov Identifier no. NCT03058367.

## Introduction

The gut microbiome is emerging as an important regulator of metabolic health and energy homeostasis (1). Derrien called the microbiota “the X-factor” that may account for the highly individualized responses to dietary interventions (2). Dietary interventions, including probiotics, prebiotics, and dietary fat, constitute one of the main strategies for modulating the gut microbiota composition and activity (3–5). Specifically, dietary fat has been shown to have a significant impact on gut microbiota whose function and clinical implication in obesity are well-documented and pose the question if dietary fat binders could contribute toward the growth of healthy gut microflora (6, 7). While studies on the microbiome and its link to obesity are compelling, we have yet to see interventions that describe significant weight loss and improved metabolic biomarkers combined with favorable shifts in the profile of the microbiota. This study describes the clinical effect of a novel formulation IQP-AE-103, consisting of a blend of two natural principal ingredients that have unique properties and can bind dietary fat synergistically in an *in vitro* setting.

The ratio of phyla Firmicutes to Bacteroidetes in mice and humans has been identified to be different in obese and lean phenotypes. Relative abundance of Firmicutes and a reduction in Bacteroidetes appears to correlate with greater efficiency in energy harvested by the microbiota and thus has been associated with the obese population (8). This study demonstrated that individuals with a high Firmicutes to Bacteroidetes ratio may extract as much as 30% more calories from the same portion of food as those with a lower ratio. This gut dysbiosis may be more likely to foster an energy net positive metabolic environment, leading to increased fat storage, greater resistance and individual variability to therapeutic interventions (9). Additionally, new research has also linked certain species or strains of organisms with leaner body types. The abundance of *Akkermansia muciniphila* is inversely associated with negative metabolic markers, including fasting glucose levels, waist-to-hip ratio, subcutaneous fat deposition, and obesity. Elevated *Akkermansia* levels in combination with greater microbial gene richness are indicative of a diverse microbiome. These elevated levels are not only more likely to be present in lean individuals, but they are also strong indicators of the subjects most likely to experience significant change with the best metabolic outcomes (10, 11). Dao showed that subjects with high levels of *A. muciniphila* at baseline had the most significant improvement in insulin sensitivity and other positive metabolic changes (12). While Dao demonstrated significant metabolic improvements after 6 weeks of a calorie-controlled diet and 6 weeks of a maintenance diet, subjects did not experience corresponding changes in weight (12). A 2019 intervention study using *A. muciniphila* similarly showed several metabolic parameters improved but did not result in statistically significant weight loss, changes in body mass index (BMI), or visceral fat deposition (11).

In a previously published, double-blind, placebo-controlled, 12-week trial, overweight and moderately obese subjects on a hypocaloric diet were treated with IQP-AE-103 (13). IQP-AE-103 is a combination of dehydrated powder of okra pods [*Abelmoschus esculentus* (L.) Moench] and inulin, a heterogeneous mixture of fructose polymers extracted from chicory roots (14, 15). This study demonstrated significant weight loss, a reduction in feelings of hunger, and a decrease in BMI, body fat mass, and waist and hip circumference as compared with the placebo (13). Much of the noted benefits were thought to be the result of the dietary fat binding by IQP-AE-103 and the physicochemical swelling and gel-forming properties of okra pods, which have been shown to induce satiety *in vivo* (13). A review of satiety-inducing polysaccharides showed that satiety was typically noted at doses of 8–10 g or more, making the satiety effect in Uebelhack’s study unique for its combined, low, daily dose of 2,490 mg (16, 17).

Carbohydrates are the most abundant macronutrient in okra pods and include both soluble and insoluble fibers (18). Okra contains highly viscous, soluble, indigestible polysaccharides, such as pectin, with strong swelling and gel-forming properties (15). Moreover, okra pods and seeds may contain several unique polyphenols, including flavonoids, isoquercitrin, and quercetin-3-O-gentiobiose (19). Okra pods contain up to 6.7% polyphenols, mostly found in the seeds (20).

In contrast, inulin is a soluble, indigestible, but non-viscous polysaccharide with less or no gel-forming properties that are rapidly fermented by the microbiota (21). Inulin and other soluble, indigestible fibers have been extensively studied for their benefits associated with glucose homeostasis, lipid regulation, colon disease prevention, and transit time, (22–30). However, clinical outcomes for inulin supplementation and weight loss are mixed. For example, Guess’s study of 30 g/day in pediatric subjects resulted in a 7.6% reduction in weight over 18 weeks (compared with 4.9% in the placebo) (31). Another study supplemented 10 g of inulin plus 10 g of resistant maltodextrin in combination with a caloric restriction and showed no significant weight loss over the placebo (32). IQP-AE-103 provided a small dose, 510 mg/day, of inulin in combination with dehydrated okra powder and showed clinically proven weight loss (13).

Deleterious gut microbiota modulation was previously thought to be a consequence of weight gain and other metabolic disturbances. However, emerging science is confirming that microbiota shifts may also contribute to causation as well as be the consequence of the hallmarks of metabolic disease (33). Studies have shown that gut microbial diversity and composition in humans is more significantly modified by dietary and/or environmental factors than genetics (34).

This study demonstrates that IQP-AE-103 leads to both a significant reduction in Firmicutes abundance and a significant increase in *A. muciniphila*, specifically in subjects who achieved at least 5% of weight loss. This shift in microbiota indicates that the fat-binding properties of inulin and okra not only exert an action on body weight but also contribute to the increase in diversity of the gut microflora.

## Materials and Methods

### Study Design and Participants

This study is a double-blind, randomized, placebo-controlled, parallel-group clinical study. It was carried out in Berlin, Germany, for over 14 weeks, including a 2-week run-in phase and a 12-week intervention phase. A total of seventy-two overweight or moderately obese Caucasian male and female volunteers (BMI ≥ 25 to < 35 kg/m^2^) aged 18–65 years participated in this study. The participants were generally healthy and fulfilled the following inclusion criteria: accustomed to three main meals daily, adherence to recommended diet, commitment to maintaining a habitual level of activity/exercise during the study, consistent and stable body weight for 3 months prior to recruitment, use of contraception methods for women of childbearing potential, and avoidance of other weight management products or programs. The main exclusion criteria of the study included hypersensitivity to any of the ingredients, pregnancy or nursing, smoking cessation within 6 months prior to recruitment or during the study, abuse of drugs and alcohol, diabetes mellitus type 1, uncontrolled diabetes mellitus type 2, uncontrolled endocrine disorders, gastrointestinal surgery within 6 months, use of any medications that may influence body weight and gastrointestinal function, acute or chronic gastrointestinal diseases, use of any electronic medical implant, serious organ or systemic diseases, and eating disorders.

Written informed consent was obtained from all the subjects before any study-related procedures were performed. This clinical study was approved by the ethics committee of Charité-University Medicine Berlin in November 2016 and was conducted according to the principles of the World Health Organization (Declaration of Helsinki) as well as the ICH guidelines and EU recommendations for Good Clinical Practice (CPMP/ICH/135/95), ICH E6). The study was registered with ClinicalTrials.gov Identifier no. NCT03058367 in February 2017. The clinical trial was initiated in April 2017 and completed in November 2017.

### Diet and Intervention

After 2 weeks of run-in phase for acclimation to the study requirements on diet and subject diary, subjects were randomly allocated to receive IQP-AE-103 (IQP) or placebo at a ratio of 1:1. The IQP group was provided with capsules each containing 330 mg of dehydrated okra powder and 85 mg of inulin as functional ingredients for a total daily dose of 1,980 and 510 mg, respectively.

The placebo group was provided with capsules containing standard excipients to replace the functional ingredients. Both the IQP and placebo capsules were identical in appearance and size. Both the IQP and placebo capsules contained excipients commonly used in the manufacturing of pharmaceutical or food products, such as diluent, flowing aid, anticaking agent, and colorant. Subjects were instructed to take two capsules, three times per day, within 15 min after each main meal (breakfast, lunch, and dinner), with a glass of water. During the intervention period, all subjects were instructed to follow a balanced and hypocaloric diet (approximately 20% deficit) consisting of 30% energy from fat. The basal daily calorie need was estimated for each subject based on sex, age, and actual body weight according to the Institute of Medicine’s equations for estimating energy requirements (35). Recommendations for a nutritionally balanced diet were created by an independent qualified nutritionist or dietician. Diet plans according to the individual’s energy requirements were provided and explained by the investigators to the subjects. An example of the diet plan is shown in Table 1.

**TABLE 1:** Example of a 1,500 kcal daily diet plan.

Energy (kcal)	Meal	Food	Portion size	Quantity (g)
400	Breakfast	Rye bread	2 slices	80
		Butter	1 tsp	5
		Gouda (45% fat in dry matter)	1 slice	30
		Chicken breast	1 slice	30
		Apple	1 medium	110
100	Snack	Yogurt (low in fat, 1.5%)	1/3 large cup	150
		Sugar	1 tsp	5
		Walnuts	1/2	2
500	Lunch	Pasta (without egg)	To weigh	70
		Cabbage	1/2 medium	250
		Cooked ham	2 slices	40
		Oil (e.g., rapeseed)	1 tsp	5
		Cream cheese (50% fat in dry matter	2 tsp	40
100	Snack	Dark chocolate	2 pieces	10
		Dried apricots	2 pieces	20
400	Dinner	Whole grain bread	2 slices	80
		Butter	1 tsp	5
		Gouda (45% fat in dry matter)	1 slice	15
		Salmon ham	3 slices	25
		Quark (20% fat in dry matter)	1 tsp	50
		Carrots	2 medium	100
		Kohlrabi	1/2 medium	100
		Kiwi	1 small	40

### Weight Loss Measurements

To investigate the effect of IQP-AE-103 supplementation on weight loss, subjects’ body weight, body composition, and waist and hip circumference were measured by trained study personnel at baseline, week 4, week 8, and week 12 of the study. Body weight and body composition were measured in subjects wearing underwear and barefoot using validated weighing scales (Tanita BC-420 MA). Waist circumference (cm) was measured at the level midway between the lateral lower rib margin and the iliac crest, whereas hip circumference (cm) was measured as the maximal circumference over the buttocks. The differences in mean body weight reduction (kg), mean body fat mass reduction (kg), and mean waist and hip circumference reduction (cm) between the IQP group and the placebo group were evaluated after 4, 8, and 12 weeks of intervention in comparison to baseline. Additionally, subgroup analysis on the same parameters on subjects who lost at least 5% of their baseline body weight, known as 5% responders (IQP5), was performed. Statistical analyses were performed using the SPSS software version 18 (Statistical Package for the Social Sciences; SPSS, Chicago, IL, United States). The non-parametric Mann-Whitney *U*-test was applied and the level of significance (*p* < 0.05) was assumed.

### Microbiome Analysis

Microbiome analysis was performed to investigate the association of changes in body weight with microbiota composition due to IQP-AE-103 supplementation. Subjects were provided with fecal collection tubes (DNA/RNA Shield™ Swab & Collection Tube, Zymo Research). The first distribution of fecal collection kits to the subjects occurred during the 2 weeks of the run-in phase (prior to the start of intervention), and the second distribution occurred between weeks 8 and 12 of the intervention phase. Subjects were instructed to collect fecal samples at week 12, based on the instructions for fecal sampling, and samples were to be returned to the investigator site within 7 days of the sample being taken. The collection tubes were stored at temperatures between −20°C and −30°C. The samples were processed and analyzed at Zymo Research (Irvine, CA, United States). DNA extraction was performed using the ZymoBIOMICS^®^-96 MagBead DNA Kit (Zymo Research, Irvine, CA, United States), with an automated platform according to the manufacturer’s instructions. Bacterial 16S ribosomal RNA gene-targeted sequencing was performed, and the V3-V4 region was amplified using the Quick-16S™ Primer Set V3-V4 (Zymo Research, Irvine, CA, United States). Real-time PCR was performed, and the final PCR products were quantified with qPCR fluorescence readings and pooled together based on equal molarity. The final pooled library was cleaned up with the Select-a-Size DNA Clean & Concentrator™ (Zymo Research, Irvine, CA), then quantified with TapeStation^®^ (Agilent Technologies, Santa Clara, CA) and Qubit™ (Thermo Fisher Scientific, Waltham, WA). The final library was sequenced on Illumina^®^ MiSeq™ with a version 3 reagent kit (600 cycles). The sequencing was performed with >10% PhiX spike-in. Unique amplicon sequences were inferred from raw reads using the DADA2 pipeline (36). Chimeric sequences were also removed with the DADA2 pipeline. Taxonomy was assigned with the Greengene 16S Research Database.

Taxonomy composition visualization and diversity analyses were performed using the Quantitative Insights Into Microbial Ecology (QIIME version 1.9.1) bioinformatics pipeline (37). A taxonomy with a statistically significant difference in abundance between the IQP and placebo groups, as well as between IQP5 and the placebo groups, was identified by linear discriminant analysis effect size (Lefse) (38).

### Statistical Analysis

Further statistical analyses were performed on differences in the relative abundance of microbiota that were linked to obesity or metabolic syndromes using the SPSS software version 18. The differences in relative abundance between the IQP and placebo groups, between the IQP5 and placebo groups, and the changes within each group were compared. The non-parametric Mann-Whitney *U*-test and Wilcoxon signed-rank test were applied and the level of significance (*p* < 0.05) was assumed.

## Results

**FIGURE 1:**
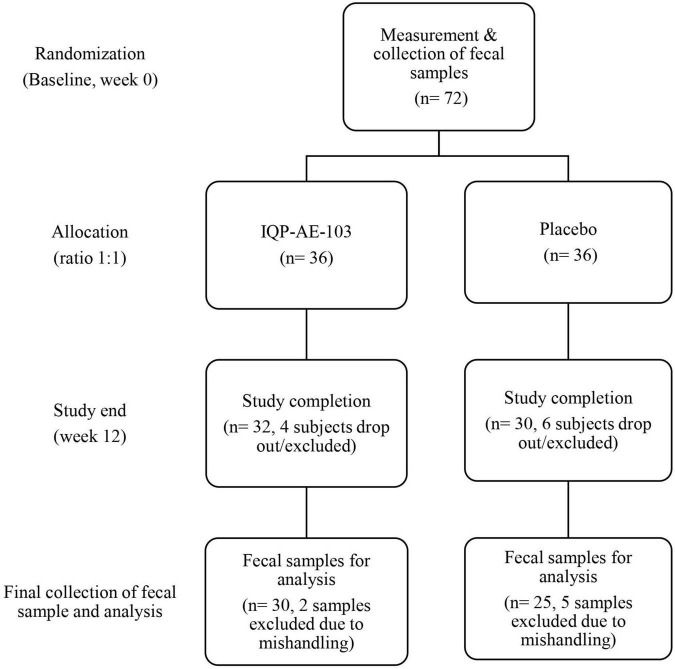
Flowchart of the study population from the start until the end of the study.

A total of 36 subjects were randomized into each study arm. Due to subjects’ dropouts or mishandling of samples, six fecal samples from the IQP group and eleven samples from the placebo group were not included for microbiome analysis (Figure 1). Thus, the fecal samples of thirty subjects in the IQP group and twenty-five subjects in the placebo group were analyzed. Due to the unavailability of fecal samples from certain subjects at baseline or at the end of the study, the intention-to-treat population analysis was not performed. The per-protocol analysis was performed only on subjects with complete fecal samples.

The mean age of the study population was 48.67 ± 11.69 years. The proportion of women to men was higher in both groups. There were no statistical differences in age, gender distribution, body height, body weight, BMI, waist circumference, hip circumference, body fat mass, fat-free mass, and energy requirements between the study groups at baseline. All subjects who completed the study met the compliance rate (≥80 to ≤120%) for intake of investigational products. Clinical baseline characteristics are summarized in Table 2.

**TABLE 2:** Baseline demographic of the study population (mean ± SD) or number (%) of participants.

Characteristics	IQP (*n* = 30)	IQP5 (*n* = 17)	Placebo (*n* = 25)
Mean age (years)	50 ± 11.9	50 ± 11.1	47 ± 11.4
Women	23 (76.7%)	12 (70.6%)	18 (72%)
Men	7 (23.3%)	5 (29.4%)	7 (28%)
Height (cm)	168.13 ± 8.99	167.88 ± 10.56	167.88 ± 10.74
Body weight (kg)	83.37 ± 10.91	85.19 ± 12.67	85.25 ± 14.54
BMI (kg/m^2^)	29.42 ± 2.27	30.11 ± 2.33	30.05 ± 2.71
Waist circumference (cm)	100.20 ± 6.71	101.71 ± 6.23	104.88 ± 11.77
Hip circumference (cm)	106.10 ± 6.62	106.59 ± 7.45	108.32 ± 6.50
Body fat mass (kg)	30.38 ± 5.92	30.58 ± 7.32	31.23 ± 7.22
Fat free mass (kg)	52.93 ± 9.67	54.50 ± 11.23	54.02 ± 11.57
Energy requirement (kcal)	2129.8 ± 334.72	2183.35 ± 375.91	2196.44 ± 419.2

*No significant differences between groups (IQP vs. placebo and IQP5 vs. placebo) for all parameters.*

After 12 weeks of intervention, body weight was significantly reduced in the IQP group compared with the placebo group, 5.16 ± 2.39 kg vs. 0.97 ± 2.09 kg (*p* < 0.001). In addition, 17 (57%) subjects in the IQP group lost at least 5% of their baseline body weight compared with only 1 (4%) subject in the placebo group. The mean body weight reduction in the subgroup of subjects who lost at least 5% body weight from the IQP group (hereafter referred to as IQP5) was 6.74 ± 1.94 kg and was significantly greater compared with the placebo group (*p* < 0.001). There were also significant differences in mean reduction of BMI, waist circumference, hip circumference, and body fat mass between the IQP and placebo groups, as well as between the IQP5 and placebo groups. Furthermore, significant differences were also observed in some of these parameters at weeks 4 and 8 between the groups. Results are summarized in Table 3.

**TABLE 3:** Mean reduction in body weight and other related parameters from baseline to week 4, week 8, and week 12, respectively (mean ± SD).

Parameters	IQP (*n* = 30)	IQP5 (*n* = 17)	Placebo (*n* = 25)	*p*-values	
				IQP vs. placebo	IQP5 vs. placebo
**Body weight reduction (kg)**					
Week 4	1.98 ± 1.39	2.28 ± 1.66	0.86 ± 1.51	**< 0.05**	**< 0.05**
Week 8	3.06 ± 1.66	3.75 ± 1.74	0.92 ± 2.00	**< 0.001**	**< 0.001**
Week 12	5.16 ± 2.39	6.74 ± 1.94	0.97 ± 2.09	**< 0.001**	**< 0.001**
**BMI reduction (kg/m^2^)**					
Week 4	0.69 ± 0.44	0.79 ± 0.52	0.30 ± 0.52	**< 0.05**	**< 0.05**
Week 8	1.07 ± 0.54	1.32 ± 0.55	0.32 ± 0.72	**< 0.001**	**< 0.001**
Week 12	1.81 ± 0.78	2.37 ± 0.55	0.33 ± 0.78	**< 0.001**	**< 0.001**
**Waist circumference reduction (cm)**					
Week 4	1.57 ± 1.72	1.47 ± 1.62	0.68 ± 1.07	0.065	0.123
Week 8	2.80 ± 2.64	3.00 ± 2.65	0.92 ± 1.29	**< 0.05**	**< 0.05**
Week 12	4.37 ± 3.33	4.59 ± 3.30	0.92 ± 1.66	**< 0.001**	**< 0.001**
**Hip circumference reduction (cm)**					
Week 4	1.20 ± 1.32	0.88 ± 1.05	0.72 ± 1.02	0.194	0.600
Week 8	2.30 ± 1.78	2.06 ± 1.56	1.04 ± 1.24	**< 0.05**	**< 0.05**
Week 12	4.07 ± 2.99	4.24 ± 3.17	1.04 ± 1.27	**< 0.001**	**< 0.001**
**Body fat mass reduction (kg)**					
Week 4	2.39 ± 5.73	2.15 ± 2.37	0.16 ± 1.84	**< 0.05**	**< 0.05**
Week 8	3.14 ± 4.77	3.63 ± 6.04	0.25 ± 2.32	**< 0.05**	**< 0.05**
Week 12	2.77 ± 2.63	3.13 ± 3.11	0.13 ± 2.95	**0.001**	**< 0.05**
**Fat-free mass reduction (kg)**					
Week 4	2.15 ± 9.43	0.02 ± 2.99	0.70 ± 2.25	0.761	0.720
Week 8	-0.13 ± 4.84	0.01 ± 6.09	0.67 ± 2.64	0.636	0.405
Week 12	2.33 ± 3.25	3.50 ± 3.59	0.84 ± 3.13	0.141	**< 0.05**

*The p-values are given in bold when significance is <0.05.*

**FIGURE 2:**
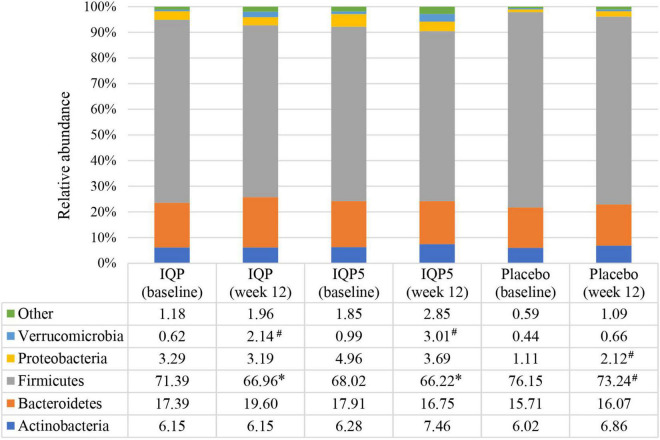
Relative abundance of each phylum at baseline and after 12 weeks is shown as a percentage (%). Pattern-filled columns of the bar chart represent the relative distribution of the five main phyla. Euryarchaeota, Cyanobacteria, Elusimicrobia, Fusobacteria, Lentisphaerae, Spirochaetes, Synergistetes, and Tenericutes are classified as “Other” due to a low abundance level of <1.0%. *p < 0.05 compared with placebo; ^#^*p* < 0.05 compared with baseline within the same groups.

The characterization of the microbiota communities present in fecal samples collected prior to the start of the intervention (week 0) and at the end of the study (week 12) was performed to investigate changes in gut microbiota composition. The relative abundances of 13 phyla were analyzed, and the five most dominant phyla (Actinobacteria, Bacteroidetes, Firmicutes, Proteobacteria, and Verrucomicrobia) are presented in Figure 2. At baseline, the placebo group had the highest relative abundance of Firmicutes (76.15 ± 8.83%), while the IQP5 group had the highest relative abundance of Bacteroidetes (17.91 ± 9.40%) and Actinobacteria (6.28 ± 5.42%), respectively. After 12 weeks, all groups had a decreased relative abundance of Firmicutes, and the placebo group had the highest relative abundance (73.24 ± 8.06%) amongst the three groups. Differences in the relative abundance of Firmicutes in the IQP group (66.96 ± 10.74%) and IQP5 group (66.22 ± 11.75%) were significant in comparison to the placebo group (*p* < 0.05). The relative abundance of Bacteroidetes and Actinobacteria was highest in the IQP (19.60 ± 9.77%) and IQP5 groups (7.46 ± 4.55%), respectively; however, the differences in comparison to the placebo group were not statistically significant. Statistical changes from baseline within groups were observed in the IQP (Verrucomicrobia), IQP5 (Verrucomicrobia), and placebo (Firmicutes and Proteobacteria) groups.

Data from the computed Firmicutes/Bacteroidetes (F/B) ratio was higher in the IQP group (19.25 ± 65.74) compared with the placebo group (9.0 ± 17.15) at baseline. In contrast, the F/B ratio of the IQP5 group (6.79 ± 7.65) was lower than the placebo group. After 12 weeks, the F/B ratio of the IQP and placebo groups were reduced to 4.54 ± 2.96 and 5.78 ± 3.41, respectively. Similarly, the F/B ratio of the IQP5 group was reduced to 5.28 ± 3.29. The F/B ratio was reduced in all three groups after 12 weeks from baseline and a distinguished reduction was observed in the IQP group; however, differences between the groups (IQP vs. placebo and IQP5 vs. placebo) were not statistically significant.

**FIGURE 3:**
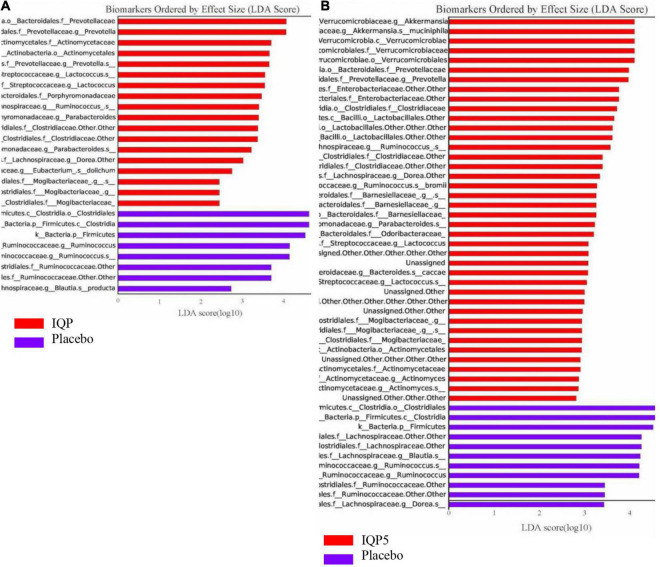
Histogram of linear discriminant analysis (LDA) scores showing the taxa abundance distribution of each microbiota classification with a statistical difference after 12 weeks. Bars in red indicate higher abundance in the IQP and IQP5 groups, while purple bars indicate higher abundance in the placebo group. **(A)** IQP group vs. placebo group; **(B)** IQP5 group vs. placebo group. Only taxa with LDA effect size > 2 are shown. f_: family; g_: genus, s_: species.

Next, Lefse analysis was performed to identify bacterial taxa distributions that are significantly and statistically different (*p* < 0.05) among the groups with an effect size [linear discriminant analysis (LDA) score] higher than 2 at different phylogenetic levels. The differences in the overall microbiota composition and abundance distribution in the IQP group vs. the placebo group and the IQP5 group vs. the placebo group are shown in the biomarker plots in Figures 3A,B. The placebo group overall had a lower microbiota composition and a less diversified microbiota community. The IQP and IQP5 groups were more enriched with several microbiota groups, for example, family Actinomycetaceae, family Mycobacteriaceae, genus *Prevotella*, genus *Lactococcus*, and genus *Akkermansia* (for the IQP5 group). In contrast, the family Lachnospiraceae and the genus *Ruminococcus* were more prevalent in the placebo group.

**FIGURE 4:**
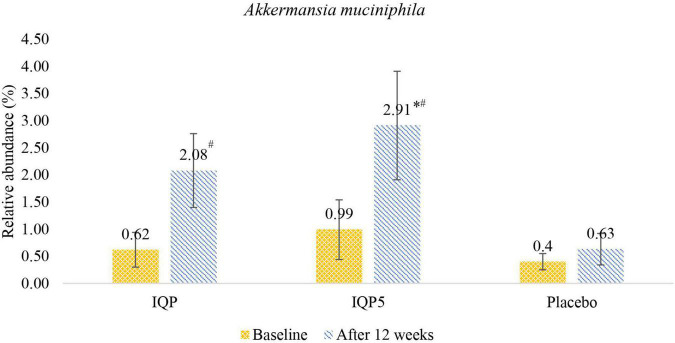
Relative abundance presented in percentage (%) of *Akkermansia muciniphila* in the IQP, IQP5, and placebo groups at baseline and after 12 weeks; *p < 0.05 compared with placebo after 12 weeks. ^#^*p* < 0.05 compared with baseline within the same groups.

Furthermore, analyses on the relative abundance of *A. muciniphila* were performed to investigate the association of the microbiota with the intake of IQP-AE-103 and effect on weight reduction. *A. muciniphila* was at least 4-fold higher in the IQP5 group than in the placebo group (2.91 ± 4.12% vs. 0.63 ± 1.43%; *p* < 0.05) after 12 weeks. No significant difference was observed in the abundance of these microbiota at baseline. Statistical changes from baseline within the group were also observed in both the IQP and IQP5 groups, but not in the placebo group (Figure 4).

In summary, the intake of IQP-AE-103 reported a significant reduction in body weight, BMI, body fat mass, waist circumference, and hip circumference. Furthermore, the IQP weight loss population had a distinct and differentiated microbiota diversity and a significant difference in the relative abundance of microorganisms such as Firmicutes and *A. muciniphila* in comparison to the placebo group.

## Discussion

This study is unique in reporting both significant weight loss and corresponding favorable shifts in Firmicutes and *A. muciniphila*, both of which are associated with better metabolic health (39–42). Similar to other studies, and in line with positive improvements in the metabolic profile, both Lefse and microbial relative abundance analyses showed that Firmicutes abundance was significantly lower in the IQP group compared with the placebo group at the end of the study (11, 12, 43).

At least 5% of weight loss was observed for 57% of subjects with IQP-AE-103 supplementation (referred to as 5% responders or IQP5). Analysis of this subgroup showed that there was also a significant increase in *A. muciniphila* of 300% over baseline and over 460% over placebo after 12 weeks. This subgroup also demonstrated a similar decrease in Firmicutes both over baseline and placebo.

While other studies investigating dietary supplementation with inulin have certainly shown a positive impact on gut microbiota, the administered dose per day of inulin has been high, ranging from 8 to 30 g, with 10 g being the average (44, 45). The dose of IQP-AE-103 in this study provided 510 mg/day of inulin. While inulin has been associated with an increase in *A. muciniphila* in both healthy and overweight and obese subjects (46), there are no other clinical studies demonstrating a reduction in weight and/or a change in *A. muciniphila* at this dose, alone or in combination with other ingredients. This low dose of inulin synergistically combined with okra powder provides similar benefits as previously reported at higher doses and without the adverse events traditionally associated with inulin, like abdominal gas and bloating or laxative effect. IQP-AE-103 had excellent tolerability, and no adverse events related to the intake of IQP-AE-103 were reported after 12 weeks of intervention (13).

Research has been accelerated on the use of *A. muciniphila* as a potential therapeutic agent for metabolic and gastrointestinal disorders, including obesity (47). The deleterious effects of a high-fat diet have been shown to be offset by *Akkermansia* enriching polyphenols, including positive modulation of intestinal gene expression, improved lipid regulation, enhanced insulin sensitivity, reduced fat deposition, and increased epithelial integrity (11, 12, 48, 49).

In 2019, the first preliminary clinical trial supplementing *Akkermansia* (live and sterilized organisms) as a targeted therapy was published. Treated subjects showed improved insulin sensitivity, reduced insulinemia and plasma, and reduced total cholesterol. Although *Akkermansia* levels showed a positive trend, the study did not achieve significant changes in body weight, fat mass, or hip and waist circumference (11). While very promising, it reinforces work previously published that confirms *A. muciniphila*’s modulation and effect are likely mediated by multiple and potentially confounding factors, including variations in diet (macronutrients as well as calories), nutritional status, host health, lumen environment, ethnicity, gender, age, medication use, mucin structure, and availability, as well as baseline abundance of *Akkermansia* and other organisms (12, 30, 50–58). These confounding factors, which contribute to modulating *Akkermansia*, may offer further explanation for many of the conflicting data noted in previous publications.

There are multiple studies in animals and humans that correlate a higher abundance of *A. muciniphila* with a lean phenotype (42, 52, 58–63). Additionally, fecal transplant studies have demonstrated that transferring the microbiota of lean or obese animals into the opposite phenotype results in the animals trending toward or adopting the metabolic state of the donor (64–68). Studies on prebiotic fibers and their impact on *Akkermansia* in humans are mixed (46, 69). While it is clear that a strong connection exists between the microbiota profile and metabolic health, there are few studies that actually demonstrate weight loss with fiber supplementation even when there is clearly a positive shift in the microbiome (11, 32, 70–72).

*Akkermansia* levels have been positively influenced in other human studies through caloric restriction, gastric bypass, and polyphenol, inulin, polydextrose, and yeast fermentate supplementation. However, with the exception of gastric bypass, none of these studies showed a concomitant change in weight or BMI (10, 57, 73–76). In type 2 diabetics, caloric restriction increased levels of *A. muciniphila* by 125% (57). Similarly, Dao showed a correlation between a reduced-calorie diet in obese and overweight subjects and an improved abundance of *A. muciniphila*. In this study, an increase in *Akkermansia* was only noted in individuals with a below median and baseline levels of the microorganism (12). Even more surprisingly, subjects with a high starting level (above the median) of *A. muciniphila* had a decrease in abundance over the course of the study.

Interestingly, Healey showed that changes in the microbiota profile were linked to dietary intervention, specifically demonstrating that subjects consuming a high fiber diet plus 16 g of inulin-type fructans/day were much more responsive to favorable shifts in their microbiota profile than those on a low fiber diet and 16 g of inulin per day. Overall, dietary records showed that the high dietary fiber group had slightly higher energy, fat, carbohydrate, and fiber intake as compared with the low dietary fiber group (77).

In this study, the protocol diet was intended to reduce calories and standardize the amount of fat intake among all the participants. While suggested menus were provided, subjects’ macronutrient consumption could vary while still meeting the caloric and fat requirements. Previous studies showed that macronutrient dietary makeup may also have a profound impact on the susceptibility of the microbiota for change, including total calories, fat, fiber, and refined carbohydrate levels (6, 7, 78). In our study, both subject groups were provided with standardized calorie and fat intake, and a positive shift in the growth of healthy gut microflora was only seen in the IQP group and more prominently in subjects with at least 5% of baseline body weight loss after taking dietary fat binder.

One of the limitations of this study could be the small sample size. The sample size was determined primarily for the weight loss effect by the Cohen’s effect size (group comparison), for the significance level of 5.0% (double-sided) and power of 80% (79). In consideration of the use of non-parametric statistical analysis, the necessary sample size was 28 subjects per study arm and was further increased to 36 subjects after consideration of dropout rate (20%) and block randomization requirements. The observed changes in the microbiome remain interesting for exploratory purposes, and the research findings warrant further study with a larger sample size.

In fact, some studies indicate that shifts in the microbiota may occur on the first day of dietary change or supplementation (80), and positive changes in the abundance of *A. muciniphila* can occur in as short as 3 days (80). Certainly, the microbiota shift occurs prior to the time point of statistically significant changes in weight loss. This supports the emerging theory that changes in the microbiota, and perhaps even specific species, may contribute to the etiology of weight changes rather than be the result of the weight changes (81).

## Conclusion

To the best of our knowledge, we report the first, controlled study in humans where an OTC supplementation resulted in both favorable changes in Firmicutes and *Akkermansia*, as well as corresponding, clinically relevant changes in weight, adiposity, and body mass index. This shift in microbiota indicates that the fat-binding properties of the synergistic blend of dehydrated okra powder and inulin not only exert an action on body weight but also on gut microflora diversity.

## Data Availability Statement

The data generated from this study is not made available to the public due to local ethical guidelines. Further inquiries can be directed to the corresponding author.

## Ethics Statement

The studies involving human participants were reviewed and approved by the Charité – University Medicine Berlin. The patients/participants provided their written informed consent to participate in this study.

## Author Contributions

LP, PD, and PC were involved in the conceptualization and design of the study. LP performed further data analyses. LP and JC wrote the manuscript. PD and PC critically reviewed and contributed to the final content of the manuscript. All authors reviewed the data and statistical analyses provided by the independent statistician, contributed to the final content, and approved the manuscript.

## Conflict of Interest

LP, PD, and PC are the employees of InQpharm Group that funded the study. InQpharm Group was not involved in the conduct of the trial or the collection of data. JC received consultation fees for the independent review of the data and contributed to the manuscript writing.

## Publisher’s Note

All claims expressed in this article are solely those of the authors and do not necessarily represent those of their affiliated organizations, or those of the publisher, the editors and the reviewers. Any product that may be evaluated in this article, or claim that may be made by its manufacturer, is not guaranteed or endorsed by the publisher.

## References

[1] Sonnenburg JL Bäckhed F. Diet-microbiota interactions as moderators of human metabolism. *Nature.* (2016) 535:56–64. 10.1038/nature18846 27383980 PMC5991619

[2] Derrien M Belzer C de Vos WM. *Akkermansia muciniphila* and its role in regulating host functions. *Microb Pathog.* (2017) 106:171–81. 10.1016/j.micpath.2016.02.005 26875998

[3] Vandeputte D Falony G Vieira-Silva S Wang J Sailer M Theis S Prebiotic inulin-type fructans induce specific changes in the human gut microbiota. *Gut.* (2017) 66:1968–74. 10.1136/gutjnl-2016-313271 28213610 PMC5739857

[4] Doestzada M Vila AV Zhernakova A Koonen DPY Weersma RK Touw DJ Pharmacomicrobiomics: a novel route towards personalized medicine? *Protein Cell.* (2018) 9:432–45. 10.1007/s13238-018-0547-2 29705929 PMC5960471

[5] Bashiardes S Godneva A Elinav E Segal E. Towards utilization of the human genome and microbiome for personalized nutrition. *Curr Opin Biotechnol.* (2018) 51:57–63. 10.1016/j.copbio.2017.11.013 29223004

[6] Cândido FG Valente FX Grześkowiak Ł M. Moreira APB Rocha DMUP Alfenas R Impact of dietary fat on gut microbiota and low-grade systemic inflammation: mechanisms and clinical implications on obesity. *Int J Food Sci Nutr.* (2018) 69:125–43. 10.1080/09637486.2017.1343286 28675945

[7] Wan Y Wang F Yuan J Li J Jiang D Zhang J Effects of dietary fat on gut microbiota and faecal metabolites, and their relationship with cardiometabolic risk factors: a 6-month randomised controlled-feeding trial. *Gut.* (2019) 68:1417–29. 10.1136/GUTJNL-2018-317609 30782617

[8] Méndez-Salazar EO Ortiz-López MG de los Granados-Silvestre MÁ Palacios-González B Menjivar M. Altered gut microbiota and compositional changes in *Firmicutes* and *Proteobacteria* in Mexican undernourished and obese children. *Front Microbiol.* (2018) 9:2494. 10.3389/FMICB.2018.02494 30386323 PMC6198253

[9] Turnbaugh PJ Hamady M Yatsunenko T Cantarel BL Duncan A Ley RE A core gut microbiome in obese and lean twins. *Nature.* (2009) 457:480–4. 10.1038/nature07540 19043404 PMC2677729

[10] Dao MC Everard A Clément K Cani PD. Losing weight for a better health: role for the gut microbiota. *Clin Nutr Exp.* (2016) 6:39–58. 10.1016/j.yclnex.2015.12.001 33094147 PMC7567023

[11] Depommier C Everard A Druart C Plovier H Van Hul M Vieira-Silva S Supplementation with *Akkermansia muciniphila* in overweight and obese human volunteers: a proof-of-concept exploratory study. *Nat Med.* (2019) 25:1096–103. 10.1038/s41591-019-0495-2 31263284 PMC6699990

[12] Dao MC Everard A Aron-Wisnewsky J Sokolovska N Prifti E Verger EO *Akkermansia muciniphila* and improved metabolic health during a dietary intervention in obesity: relationship with gut microbiome richness and ecology. *Gut.* (2016) 65:426–36. 10.1136/gutjnl-2014-308778 26100928

[13] Uebelhack R Bongartz U Seibt S Bothe G Chong PW De Costa P Double-blind, randomized, three-armed, placebo-controlled, clinical investigation to evaluate the benefit and tolerability of two dosages of IQP-AE-103 in reducing body weight in overweight and moderately obese subjects. *J Obes.* (2019) 2019:3412952. 10.1155/2019/3412952 30863632 PMC6377993

[14] Makhadmeh IM Ereifej KI. Geometric characteristics and chemical composition of okra (*Hibiscus esculentus* L.) grown under semi-arid conditions. *Int J Food Prop.* (2004) 7:83–90. 10.1081/jfp-120022983

[15] Bakre L Jaiyeoba K. Studies on the physicochemical properties of *Abelmuscus esculentus* L. (okra) pods – a potential tablet excipient. *Int J Biol Chem Sci.* (2009) 3:448–56. 10.4314/ijbcs.v3i3.45345

[16] Wanders AJ Mars M Borgonjen-van den Berg KJ de Graaf C Feskens EJ. Satiety and energy intake after single and repeated exposure to gel-forming dietary fiber: post-ingestive effects. *Int J Obes.* (2014) 38:794–800. 10.1038/ijo.2013.176 24030518

[17] Forde CG Almiron-Roig E Brunstrom JM. Expected satiety: application to weight management and understanding energy selection in humans. *Curr Obes Rep.* (2015) 4:131–40. 10.1007/s13679-015-0144-0 26627096 PMC4881812

[18] Petropoulos S Fernandes Â Barros L Ferreira I. Chemical composition, nutritional value and antioxidant properties of Mediterranean okra genotypes in relation to harvest stage. *Food Chem.* (2018) 242:466–74. 10.1016/j.foodchem.2017.09.082 29037716

[19] Arapitsas P. Identification and quantification of polyphenolic compounds from okra seeds and skins. *Food Chem.* (2008) 110:1041–5. 10.1016/j.foodchem.2008.03.014 26047300

[20] Xia F Zhong Y Li M Chang Q Liao Y Liu X Antioxidant and anti-fatigue constituents of okra. *Nutrients.* (2015) 7:8846–58. 10.3390/nu7105435 26516905 PMC4632455

[21] Raninen K Lappi J Mykkänen H Poutanen K. Dietary fiber type reflects physiological functionality: comparison of grain fiber, inulin, and polydextrose. *Nutr Rev.* (2011) 69:9–21. 10.1111/j.1753-4887.2010.00358.x 21198631

[22] Reis SA Conceição LL Rosa DD Dias MM Peluzio Mdo C. Mechanisms used by inulin-type fructans to improve the lipid profile. *Nutr Hosp.* (2014) 31:528–34. 10.3305/nh.2015.31.2.7706 25617533

[23] Falony G Joossens M Vieira-Silva S Wang J Darzi Y Faust K Population-level analysis of gut microbiome variation. *Science.* (2016) 352:560–4. 10.1126/science.aad3503 27126039

[24] Holscher HD Bauer LL Gourineni V Pelkman CL Fahey GC Jr Swanson KS. Agave inulin supplementation affects the fecal microbiota of healthy adults participating in a randomized, double-blind, placebo-controlled, crossover trial. *J Nutr.* (2015) 145:2025–32. 10.3945/jn.115.217331 26203099

[25] Macfarlane GT Macfarlane S. Fermentation in the human large intestine: its physiologic consequences and the potential contribution of prebiotics. *J Clin Gastroenterol.* (2011) 45(Suppl. 3):S120–7. 10.1097/MCG.0b013e31822fecfe 21992950

[26] Rebello CJ Burton J Heiman M Greenway FL. Gastrointestinal microbiome modulator improves glucose tolerance in overweight and obese subjects: a randomized controlled pilot trial. *J Diabetes Complications.* (2015) 29:1272–6. 10.1016/j.jdiacomp.2015.08.023 26424589 PMC4656110

[27] Tropini C Moss EL Merrill BD Ng KM Higginbottom SK Casavant EP Transient osmotic perturbation causes long-term alteration to the gut microbiota. *Cell.* (2018) 173:1742–54.e17. 10.1016/j.cell.2018.05.008 29906449 PMC6061967

[28] Vandeputte D Falony G Vieira-Silva S Tito RY Joossens M Raes J. Stool consistency is strongly associated with gut microbiota richness and composition, enterotypes and bacterial growth rates. *Gut.* (2016) 65:57–62. 10.1136/gutjnl-2015-309618 26069274 PMC4717365

[29] Weitkunat K Schumann S Petzke KJ Blaut M Loh G Klaus S. Effects of dietary inulin on bacterial growth, short-chain fatty acid production and hepatic lipid metabolism in gnotobiotic mice. *J Nutr Biochem.* (2015) 26:929–37. 10.1016/j.jnutbio.2015.03.010 26033744

[30] Zhang Q Yu H Xiao X Hu L Xin F Yu X. Inulin-type fructan improves diabetic phenotype and gut microbiota profiles in rats. *PeerJ.* (2018) 6:e4446. 10.7717/peerj.4446 29507837 PMC5835350

[31] Guess ND Dornhorst A Oliver N Bell JD Thomas EL Frost GS. A randomized controlled trial: the effect of inulin on weight management and ectopic fat in subjects with prediabetes. *Nutr Metab (Lond).* (2015) 12:36. 10.1186/s12986-015-0033-2 26500686 PMC4619305

[32] Hess AL Benítez-Páez A Blædel T Larsen LH Iglesias JR Madera C The effect of inulin and resistant maltodextrin on weight loss during energy restriction: a randomised, placebo-controlled, double-blinded intervention. *Eur J Nutr.* (2020) 59:2507–24. 10.1007/s00394-019-02099-x 31605197

[33] Zhao L. The gut microbiota and obesity: from correlation to causality. *Nat Rev Microbiol.* (2013) 11:639–47. 10.1038/nrmicro3089 23912213

[34] De Filippo C Cavalieri D Di Paola M Ramazzotti M Poullet JB Massart S Impact of diet in shaping gut microbiota revealed by a comparative study in children from Europe and rural Africa. *Proc Natl Acad Sci USA.* (2010) 107:14691–6. 10.1073/pnas.1005963107 20679230 PMC2930426

[35] Institute of Medicine. *Dietary Reference Intakes for Energy, Carbohydrate, Fiber, Fat, Fatty Acids, Cholesterol, Protein, and Amino Acids.* Washington, DC: The National Academies Press (2005). 10.17226/10490

[36] Callahan BJ McMurdie PJ Rosen MJ Han AW Johnson AJ Holmes SP. DADA2: high-resolution sample inference from Illumina amplicon data. *Nat Methods.* (2016) 13:581–3. 10.1038/nmeth.3869 27214047 PMC4927377

[37] Caporaso JG Kuczynski J Stombaugh J Bittinger K Bushman FD Costello EK QIIME allows analysis of high-throughput community sequencing data. *Nat Methods.* (2010) 7:335–6. 10.1038/nmeth.f.303 20383131 PMC3156573

[38] Segata N Izard J Waldron L Gevers D Miropolsky L Garrett WS Metagenomic biomarker discovery and explanation. *Genome Biol.* (2011) 12:R60. 10.1186/gb-2011-12-6-r60 21702898 PMC3218848

[39] Ley RE Turnbaugh PJ Klein S Gordon JI. Microbial ecology: human gut microbes associated with obesity. *Nature.* (2006) 444:1022–3. 10.1038/4441022a 17183309

[40] Larsen N Vogensen FK van den Berg FW Nielsen DS Andreasen AS Pedersen BK Gut microbiota in human adults with type 2 diabetes differs from non-diabetic adults. *PLoS One.* (2010) 5:e9085. 10.1371/journal.pone.0009085 20140211 PMC2816710

[41] Xu Y Wang N Tan HY Li S Zhang C Feng Y. Function of *Akkermansia muciniphila* in obesity: interactions with lipid metabolism, immune response and gut systems. *Front Microbiol.* (2020) 11:219. 10.3389/fmicb.2020.00219 32153527 PMC7046546

[42] Everard A Belzer C Geurts L Ouwerkerk JP Druart C Bindels LB Cross-talk between *Akkermansia muciniphila* and intestinal epithelium controls diet-induced obesity. *Proc Natl Acad Sci USA.* (2013) 110:9066–71. 10.1073/pnas.1219451110 23671105 PMC3670398

[43] Plovier H Everard A Druart C Depommier C Van Hul M Geurts L A purified membrane protein from *Akkermansia muciniphila* or the pasteurized bacterium improves metabolism in obese and diabetic mice. *Nat Med.* (2017) 23:107–13. 10.1038/nm.4236 27892954

[44] Wang L Yang H Huang H Zhang C Zuo H-X Xu P Inulin-type fructans supplementation improves glycemic control for the prediabetes and type 2 diabetes populations: results from a GRADE-assessed systematic review and dose–response meta-analysis of 33 randomized controlled trials. *J Transl Med.* (2019) 17:410. 10.1186/S12967-019-02159-0 31805963 PMC6896694

[45] Watanabe M Risi R Masi D Caputi A Balena A Rossini G Current evidence to propose different food supplements for weight loss: a comprehensive review. *Nutrients.* (2020) 12:2873. 10.3390/NU12092873 32962190 PMC7551574

[46] Verhoog S Taneri PE Roa Díaz ZM Marques-Vidal P Troup JP Bally L Dietary factors and modulation of bacteria strains of *Akkermansia muciniphila* and *Faecalibacterium prausnitzii*: a systematic review. *Nutrients.* (2019) 11:1565. 10.3390/nu11071565 31336737 PMC6683038

[47] Macchione IG Lopetuso LR Ianiro G Napoli M Gibiino G Rizzatti G *Akkermansia muciniphila*: key player in metabolic and gastrointestinal disorders. *Eur Rev Med Pharmacol Sci.* (2019) 23:8075–83. 10.26355/eurrev_201909_19024 31599433

[48] Baldwin J Collins B Wolf PG Martinez K Shen W Chuang CC Table grape consumption reduces adiposity and markers of hepatic lipogenesis and alters gut microbiota in butter fat-fed mice. *J Nutr Biochem.* (2016) 27:123–35. 10.1016/j.jnutbio.2015.08.027 26423887 PMC4933288

[49] Cani PD de Vos WM. Next-generation beneficial microbes: the case of *Akkermansia muciniphila*. *Front Microbiol.* (2017) 8:1765. 10.3389/fmicb.2017.01765 29018410 PMC5614963

[50] Biagi E Franceschi C Rampelli S Severgnini M Ostan R Turroni S Gut microbiota and extreme longevity. *Curr Biol.* (2016) 26:1480–5. 10.1016/j.cub.2016.04.016 27185560

[51] Collado MC Derrien M Isolauri E de Vos WM Salminen S. Intestinal integrity and *Akkermansia muciniphila*, a mucin-degrading member of the intestinal microbiota present in infants, adults, and the elderly. *Appl Environ Microbiol.* (2007) 73:7767–70. 10.1128/aem.01477-07 17933936 PMC2168041

[52] Karlsson CL Onnerfält J Xu J Molin G Ahrné S Thorngren-Jerneck K. The microbiota of the gut in preschool children with normal and excessive body weight. *Obesity (Silver Spring).* (2012) 20:2257–61. 10.1038/oby.2012.110 22546742

[53] Griffin NW Ahern PP Cheng J Heath AC Ilkayeva O Newgard CB Prior dietary practices and connections to a human gut microbial metacommunity alter responses to diet interventions. *Cell Host Microbe.* (2017) 21:84–96. 10.1016/j.chom.2016.12.006 28041931 PMC5234936

[54] Belkaid Y Hand TW. Role of the microbiota in immunity and inflammation. *Cell.* (2014) 157:121–41. 10.1016/j.cell.2014.03.011 24679531 PMC4056765

[55] Walker JM Eckardt P Aleman JO da Rosa JC Liang Y Iizumi T The effects of trans-resveratrol on insulin resistance, inflammation, and microbiota in men with the metabolic syndrome: a pilot randomized, placebo-controlled clinical trial. *J Clin Transl Res.* (2019) 4:122–35. 30873501 PMC6412609

[56] Fava F Gitau R Griffin BA Gibson GR Tuohy KM Lovegrove JA. The type and quantity of dietary fat and carbohydrate alter faecal microbiome and short-chain fatty acid excretion in a metabolic syndrome ‘at-risk’ population. *Int J Obes.* (2013) 37:216–23. 10.1038/ijo.2012.33 22410962

[57] Medina-Vera I Sanchez-Tapia M Noriega-López L Granados-Portillo O Guevara-Cruz M Flores-López A A dietary intervention with functional foods reduces metabolic endotoxaemia and attenuates biochemical abnormalities by modifying faecal microbiota in people with type 2 diabetes. *Diabetes Metab.* (2019) 45:122–31. 10.1016/j.diabet.2018.09.004 30266575

[58] Derrien M van Passel MW van de Bovenkamp JH Schipper RG de Vos WM Dekker J. Mucin-bacterial interactions in the human oral cavity and digestive tract. *Gut Microbes.* (2010) 1:254–68. 10.4161/gmic.1.4.12778 21327032 PMC3023607

[59] Png CW Lindén SK Gilshenan KS Zoetendal EG McSweeney CS Sly LI Mucolytic bacteria with increased prevalence in IBD mucosa augment in vitro utilization of mucin by other bacteria. *Am J Gastroenterol.* (2010) 105:2420–8. 10.1038/ajg.2010.281 20648002

[60] Santacruz A Collado MC García-Valdés L Segura MT Martín-Lagos JA Anjos T Gut microbiota composition is associated with body weight, weight gain and biochemical parameters in pregnant women. *Br J Nutr.* (2010) 104:83–92. 10.1017/s0007114510000176 20205964

[61] Teixeira T Grześkowiak LM Salminen S Laitinen K Bressan J Gouveia Peluzio M. Faecal levels of *Bifidobacterium* and *Clostridium coccoides* but not plasma lipopolysaccharide are inversely related to insulin and HOMA index in women. *Clin Nutr.* (2013) 32:1017–22. 10.1016/j.clnu.2013.02.008 23538004

[62] Zhang X Shen D Fang Z Jie Z Qiu X Zhang C Human gut microbiota changes reveal the progression of glucose intolerance. *PLoS One.* (2013) 8:e71108. 10.1371/journal.pone.0071108 24013136 PMC3754967

[63] Zhang T Li Q Cheng L Buch H Zhang F. *Akkermansia muciniphila* is a promising probiotic. *Microb Biotechnol.* (2019) 12:1109–25. 10.1111/1751-7915.13410 31006995 PMC6801136

[64] Bäckhed F Ding H Wang T Hooper LV Koh GY Nagy A The gut microbiota as an environmental factor that regulates fat storage. *Proc Natl Acad Sci USA.* (2004) 101:15718–23. 10.1073/pnas.0407076101 15505215 PMC524219

[65] Turnbaugh PJ Ley RE Mahowald MA Magrini V Mardis ER Gordon JI. An obesity-associated gut microbiome with increased capacity for energy harvest. *Nature.* (2006) 444:1027–31. 10.1038/nature05414 17183312

[66] Grigorescu I Dumitrascu DL. Implication of gut microbiota in diabetes mellitus and obesity. *Acta Endocrinol (Buchar).* (2016) 12:206–14. 10.4183/aeb.2016.206 31149088 PMC6535288

[67] Le Chatelier E Nielsen T Qin J Prifti E Hildebrand F Falony G Richness of human gut microbiome correlates with metabolic markers. *Nature.* (2013) 500:541–6. 10.1038/nature12506 23985870

[68] Lee H Lee Y Kim J An J Lee S Kong H Modulation of the gut microbiota by metformin improves metabolic profiles in aged obese mice. *Gut Microbes.* (2018) 9:155–65. 10.1080/19490976.2017.1405209 29157127 PMC5989809

[69] Geerlings SY Kostopoulos I de Vos WM Belzer C. *Akkermansia muciniphila* in the human gastrointestinal tract: when, where, and how? *Microorganisms.* (2018) 6:75. 10.3390/microorganisms6030075 30041463 PMC6163243

[70] Ramirez-Farias C Slezak K Fuller Z Duncan A Holtrop G Louis P. Effect of inulin on the human gut microbiota: stimulation of *Bifidobacterium adolescentis* and *Faecalibacterium prausnitzii*. *Br J Nutr.* (2008) 101:541–50. 10.1017/s0007114508019880 18590586

[71] Scott KP Martin JC Duncan SH Flint HJ. Prebiotic stimulation of human colonic butyrate-producing bacteria and bifidobacteria, in vitro. *FEMS Microbiol Ecol.* (2014) 87:30–40. 10.1111/1574-6941.12186 23909466

[72] Roshanravan N Mahdavi R Alizadeh E Jafarabadi MA Hedayati M Ghavami A Effect of butyrate and inulin supplementation on glycemic status, lipid profile and glucagon-like peptide 1 level in patients with type 2 diabetes: a randomized double-blind, placebo-controlled trial. *Horm Metab Res.* (2017) 49:886–91. 10.1055/s-0043-119089 28962046

[73] Pinheiro I Robinson L Verhelst A Marzorati M Winkens B den Abbeele PV A yeast fermentate improves gastrointestinal discomfort and constipation by modulation of the gut microbiome: results from a randomized double-blind placebo-controlled pilot trial. *BMC Complement Altern Med.* (2017) 17:441. 10.1186/s12906-017-1948-0 28870194 PMC5584023

[74] Most J Tosti V Redman LM Fontana L. Calorie restriction in humans: an update. *Ageing Res Rev.* (2017) 39:36–45. 10.1016/j.arr.2016.08.005 27544442 PMC5315691

[75] Anhê FF Roy D Pilon G Dudonné S Matamoros S Varin TV A polyphenol-rich cranberry extract protects from diet-induced obesity, insulin resistance and intestinal inflammation in association with increased *Akkermansia* spp. population in the gut microbiota of mice. *Gut.* (2015) 64:872–83. 10.1136/gutjnl-2014-307142 25080446

[76] Anhê FF Pilon G Roy D Desjardins Y Levy E Marette A. Triggering *Akkermansia* with dietary polyphenols: a new weapon to combat the metabolic syndrome? *Gut Microbes.* (2016) 7:146–53. 10.1080/19490976.2016.1142036 26900906 PMC4856456

[77] Healey G Murphy R Butts C Brough L Whelan K Coad J. Habitual dietary fibre intake influences gut microbiota response to an inulin-type fructan prebiotic: a randomised, double-blind, placebo-controlled, cross-over, human intervention study. *Br J Nutr.* (2018) 119:176–89. 10.1017/S0007114517003440 29307330

[78] Leeming ER Johnson AJ Spector TD Le Roy CI. Effect of diet on the gut microbiota: rethinking intervention duration. *Nutrients.* (2019) 11:2862. 10.3390/nu11122862 31766592 PMC6950569

[79] Grube B Chong PW Lau KZ Orzechowski HD. A natural fiber complex reduces body weight in the overweight and obese: a double-blind, randomized, placebo-controlled study. *Obesity (Silver Spring).* (2013) 21:58–64. 10.1002/oby.20244 23505169 PMC3627296

[80] David LA Maurice CF Carmody RN Gootenberg DB Button JE Wolfe BE Diet rapidly and reproducibly alters the human gut microbiome. *Nature.* (2014) 505:559–63. 10.1038/nature12820 24336217 PMC3957428

[81] Sanna S van Zuydam NR Mahajan A Kurilshikov A Vich Vila A Võsa U Causal relationships among the gut microbiome, short-chain fatty acids and metabolic diseases. *Nat Genet.* (2019) 51:600–5. 10.1038/s41588-019-0350-x 30778224 PMC6441384

